# Association of the child opportunity index with in-hospital mortality and persistence of organ dysfunction at one week after onset of Phoenix Sepsis among children admitted to the pediatric intensive care unit with suspected infection

**DOI:** 10.1371/journal.pdig.0000763

**Published:** 2025-04-14

**Authors:** Ronald Moore, Daniela Chanci, Stephanie R Brown, Michael J Ripple, Natalie R Bishop, Jocelyn Grunwell, Rishikesan Kamaleswaran

**Affiliations:** 1 Department of Surgery, Duke University School of Medicine, Durham, North Carolina, United States of America; 2 Department of Biomedical Engineering, Duke University, Durham, North Carolina, United States of America; 3 Department of Pediatrics, Emory University School of Medicine, Atlanta, Georgia, United States of America; 4 Division of Critical Care Medicine, Children’s Healthcare of Atlanta, Atlanta, Georgia, United States of America; Chinese PLA General Hospital, CHINA

## Abstract

The social determinants of health (SDoH) are fundamental factors that contribute to overall health and health-related outcomes. Children living in lower socioeconomic areas have a higher risk of critical illness and worse outcomes compared to children living in more socioeconomically advantaged areas. In this work, we determine whether the Child Opportunity Index (COI 3.0), a multi-dimensional child-specific indicator of neighborhood environment, is associated with in-hospital mortality or persistence of a Phoenix Sepsis Score  ≥  2 at one week following Phoenix Sepsis onset in children admitted to pediatric intensive care units (PICUs) with suspected infection. We performed a retrospective cohort analysis of 63,824 patients with suspected or confirmed infection admission diagnosis in two PICUs in Atlanta, Georgia with a Georgia residential address that could be geocoded and linked to a census tract. The primary outcome was the composite of in-hospital mortality or persistence of a Phoenix Sepsis Score  ≥  2 at one week following Phoenix Sepsis onset. Model performance measures of interest were the area under the receiver operating characteristic curve (AUROC) and the area under the precision-recall curve (AUPRC). Models developed with electronic medical record (EMR) data using Egleston (EG) or Scottish Rite (SR) as the training site achieved AUROCs of 0.81–0.84 (95% CI range: 0.8–0.85) and 0.82–0.82 (95% CI range: 0.81–0.83) and AUPRCs of 0.59–0.68 (95% CI range: 0.58–0.69) and 0.62–0.64 (95% CI range: 0.61–0.65) respectively. Despite significant differences in COI 3.0 characteristics and overall in-hospital mortality of children with Phoenix suspected infection between the EG and SR PICUs, the addition of COI 3.0 did not improve the overall model performance metrics. While children admitted to both PICUs were more often from COI 3.0 neighborhoods in the lowest two quintiles, these neighborhood features had less of an impact on the model’s predictive performance compared to patient physiologic and biologic features available in the EMR.

## Author summary

The social determinants of health (SDoH) are fundamental factors that contribute to overall health and health-related outcomes. Children in lower socioeconomic areas are at greater risk of critical illness or worse outcomes compared to those in more socioeconomically advantaged areas. Therefore, the inclusion of SDoH information into model development may lead to improved performance in clinical risk prediction models. In this work, we investigate whether the Child Opportunity Index (COI 3.0), a multi-dimensional child-specific indicator of neighborhood environment developed using the SDoH, is associated with in-hospital mortality or persistence of a Phoenix Sepsis Score  ≥  2 at one week following Phoenix Sepsis onset in children admitted to pediatric intensive care units (PICUs) with suspected infection. Our results show that despite differences in COI 3.0 characteristics and in-hospital mortality between the two PICUs, the addition of COI 3.0 variables did not improve the overall model performance. These findings suggest that the association of COI 3.0 variables with different outcomes, such as readmission, needs to be investigated further.

## Introduction

Health inequities in the delivery of healthcare and in outcomes are strongly associated with increased use of emergency medical services and hospitalization, including pediatric intensive care unit (PICU) admissions, revisits for acute ambulatory conditions, and hospital mortality [1–12]. Social determinants of health (SDoH) are basic social and structural factors in a person’s environment that affect their development, health, well-being, functioning, and quality-of-life outcomes (https://health.gov/healthypeople/priority-areas/social-determinants-health). The Centers for Disease Control and Prevention (CDC) identify five SDoH domains (economic stability, education access and quality, neighborhoods and the built environment, health care access and quality, and social and community) that account for the majority of modifiable factors contributing to disparate health outcomes [13,14]. Historically, unmodifiable factors such as race, ethnicity, and health insurance status have been used as proxies for the SDoH. Rather than using race or ethnicity as a substitution for the SDoH, the Child Opportunity Index (COI) quantifies the relative composite of neighborhood (census tract) SDoH metrics within the environmental, educational, and socioeconomic conditions for nearly every census tract in the U.S. Although the COI was recently updated to its third iteration (COI 3.0) [15] with the addition of variables, the COI 2.0 has been applied to children admitted to 15 PICUs across the U.S. participating in the Virtual Pediatric Systems (VPS) from January 2019 to December 2020 who had an index admission for sepsis [1].

In our prior work, we derived and validated machine learning models predicting in-hospital mortality for children admitted to either of the two Children’s Healthcare of Atlanta PICUs who met Phoenix sepsis criteria. We noted marked differences in demographic features and in-hospital mortality outcomes in children with sepsis between the two campus PICUs. As a result, we wondered whether neighborhood-level SDoH factors played a role in differences in sepsis-related outcomes, including a composite primary outcome of in-hospital mortality or persistence of  ≥  2 organ failures at one week following Phoenix sepsis onset. We hypothesized that incorporation of both individual and composite COI 3.0 measures would improve model performance when tested on the other site.

## Methods

### Ethics statement

The Children’s Healthcare of Atlanta (Children’s) Institutional Review Board (IRB) approved this study (STUDY00000483, modification approved on 2/7/2022) with a waiver of informed consent in accordance with the ethical standards of the Helsinki Declaration of 1975.

### Study design, setting, and population

**Fig 1:**
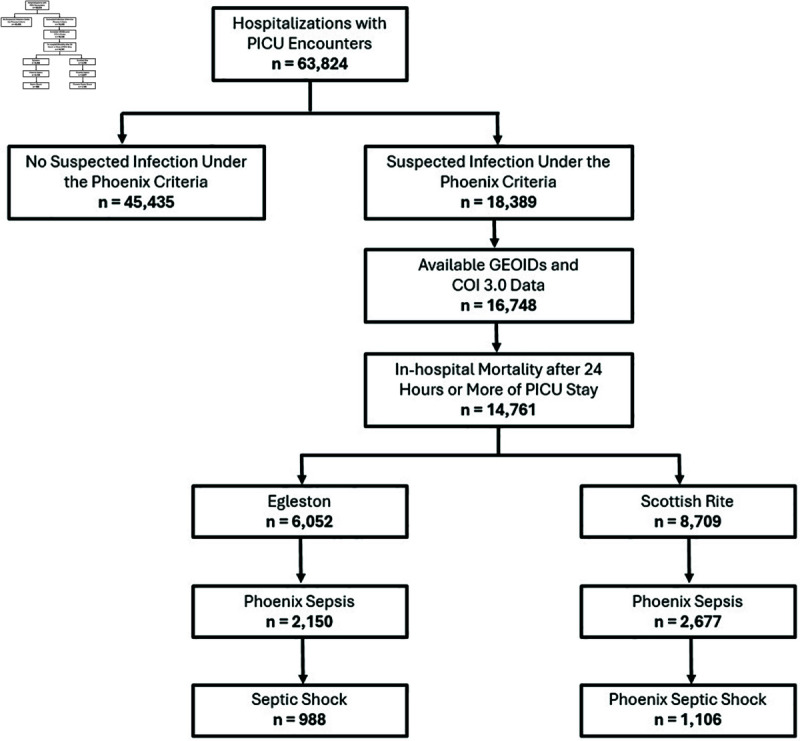
Flow diagram of the study cohort. The GEOID is the 11-digit geographical identification number for a given census tract. The COI 3.0 is the third iteration of the Child Opportunity Index.

This was a retrospective cohort study performed using children  ≤  17 years of age admitted to a Children’s PICU with suspected infection within 24 hours of the encounter between January 1, 2010 to May 10, 2022. The Children’s Healthcare of Atlanta system has two independently staffed PICUs. The Egleston (EG) PICU is a 36-bed university-affiliated, quaternary care unit with advanced technology capability (extra-corporeal life support [ECLS] and continuous renal replacement therapy [CRRT]). Children who receive hematopoietic stem cell transplants and those with unrepaired congenital heart disease are only cared for at Egleston. The Scottish Rite (SR) PICU is a 56-bed community, tertiary care unit with capability for CRRT. Since the SR PICU does not have ELCS capabilities, patients are sometimes transferred to the EG PICU for possible cannulation to ELCS. Both sites were reciprocally tested as the derivation and internal validation sites and as the external validation sites. Children readmitted to the PICU were included as a separate encounter if they had been discharged and the subsequent admission was a new, distinct hospital encounter. Children were excluded from the study if they died within 24-hours of PICU admission or if they had no available data in the 24 hours of the encounter (for characteristics of the excluded cohort, see S7 Table). The study flow diagram is available in Fig 1.

### Geocoding

Residential addresses were geocoded to census tracts using the United States (U.S.) Census Bureau batch address geographies web-based services mapped to the American Community Survey (ACS) 2019 census tract geographic identifiers (GEOIDs) (https://geocoding.geo.census.gov/geocoder/geographies/addressbatch?form). Addresses that could not be matched were geocoded using geographic coordinates with the U.S. Census Bureau’s web-based tool (https://geocoding.geo.census.gov/geocoder/geographies/coordinates). The U.S. Census 2019 GEOIDs (same as the 2010 census tract GEOIDs) for each census tract in Georgia were obtained using the R programming language [16] and the R package tigris [17]. The 2020 COI 3.0 variables were joined to patient data using the 2010 census tract GEOID [18] using the R package tidyverse [19]. The COI 3.0 consists of 44 indicators, 14 z-score normalized subdomains (early childhood education, elementary education, secondary and post-secondary education, education resources, pollution, healthy environments, safety- and health-related resources, economic opportunities, economic resources, concentrated inequity, housing resources, social resources, and wealth), and 3 domains (education, health and environment, social and economic) that are available annually for years 2012-2021 at the census tract level for regionally normed national, state, and metro areas [15]. Measures of racial and ethnic composition are intentionally excluded. The year 2020 state-based rankings for Georgia were used in the analysis.

### Outcomes, definitions, and main measures

Children with suspected infection were defined as having received antibiotics administered within the first 24-hours of hospital admission or antibiotics administered along with an order for any microbiological culture within the same 24-hour window [20,21]. Pediatric sepsis was defined as a Phoenix Sepsis Score of  ≥  2 points in a child with suspected infection. Septic shock was defined as a Phoenix Sepsis Score of  ≥  2 points with at least 1 point in the cardiovascular domain. The code used to define the cohort and build the model is warehoused at https://github.com/dchancia/ped-sepsis-prediction-ml.

The primary outcome was a composite outcome of in-hospital mortality or persistence of  ≥  2 organ failures at one week or more following Phoenix sepsis onset. In-hospital mortality was defined as mortality in the hospital within 28 days of PICU stay. Demographic data, vital signs, laboratory values, medications, and illness severity scores were used as predictor variables. We abstracted all demographic and clinical data within a 24-hour window into a clinical data repository from the EMR within the first 24-hours of the encounter. Dynamic features such as vital signs and laboratory measurements were also included. Calculated values, such as the pediatric Sequential Organ Failure Assessment (pSOFA) and the PaO2/FiO2 ratio, as well as binary indicators of medication administration and abnormal clinical variables such as heart rate and WBC were included as previously published (see S1 Table) [22]. We also used the COI 3.0 features described in Table 2.

### Data preprocessing and aggregation

Data preprocessing and aggregation were performed as previously described in Chanci et al [22]. Briefly, we removed extreme outliers defined as values > 99th or < 1st percentiles. The median value was used if multiple measurements were available. To handle missing values, we carried forward the last recorded value in a sample-and-hold interpolation pattern for any vital sign up to 12 hours prior and for any laboratory test up to 36 hours prior to the missing value for a patient-wise imputation. We used the population median to impute any remaining missing values. Finally, we aggregated the vital signs and laboratory values for each 24-hour period by calculating the mean, median, minimum, maximum, and standard deviation such that each dynamic feature was converted into a stationary feature. Feature values were then standardized by removing the mean and scaling to unit variance. Categorical features were encoded as integer values. COI 3.0 features were scaled to a value range between 0 and 1, inclusive.

### Models

We trained an eXtreme gradient boosting (XGB) model [23] to predict the composite primary outcome using the preprocessed variables. The data preprocessing, model training, and validation pipeline is shown in S1 Fig. We developed different models using only EMR features (228 features), COI features (18 features), or both EMR and COI features (246 features) and using EG as the derivation site or SR as the derivation site, resulting in a total of 6 models. For each model, we used 70% of the derivation site data to train the model and 30% as a hold-out dataset to internally validate the model. Then we used all of the validation site data for external validation. We identified the optimal hyperparameters using the 70% train dataset using 5-fold cross-validation. We randomly generated a 70/30 split of the derivation dataset using 10 different seeds with the Scikit-learn (sklearn) library [24] to calculate the 95% confidence intervals (CI) for the internal validation. To balance the number of observations between the majority class and the minority class in the train dataset, we used the Synthetic Minority Oversampling Technique (SMOTE) [25]. SMOTE uses observations from the minority class to randomly generate a new synthetic sample that has similar feature values to those observed in the minority class. The prediction threshold for each model was determined by fixing the sensitivity (recall) at 85%. At this threshold, we observed the model performance in terms of accuracy, precision (positive predictive value [PPV]), and specificity. Additionally, we compared the F1-score, area under the receiver operating characteristic curve (AUROC), area under the precision-recall curve (AUPRC), and calibration of the XGB models.

### Model interpretability

We generated Shapley beeswarm and scatter plots of the most important XGB model features using the SHapley Additive exPlanations (SHAP) package in Python [26].

**Table 1:** Cohort characteristics for pediatric patients admitted to the ICU from 2010 to 2022.

Characteristic	Total encounters	EG	SR	p-value^a^
	n = 14761 (100%)	n = 6052 (41%)	n = 8709 (59%)	
Age (years), median [Q1,Q3]	3.7 [1.0,10.6]	4.3 [1.1,11.4]	3.4 [0.9,10.0]	<0.001
Age Group, n (%)				
≤ 28 days	621 (4.2)	196 (3.2)	425 (4.9)	<0.001
29 days - 2 years	6190 (41.9)	2439 (40.3)	3751 (43.1)	<0.001
3 - 5 years	2035 (13.8)	839 (13.9)	1196 (13.7)	
6 - 17 years	5915 (40.1)	2578 (42.6)	3337 (38.3)	
Sex, n (%)				
Female	6605 (44.7)	2681 (44.3)	3924 (45.1)	0.372
Male	8156 (55.3)	3371 (55.7)	4785 (54.9)	
Race, n (%)				
White or Caucasian	6442 (43.6)	2247 (37.1)	4195 (48.2)	<0.001
Black or African American	5702 (38.6)	3067 (50.7)	2635 (30.3)	
Asian	525 (3.6)	171 (2.8)	354 (4.1)	
Other or Unknown	2092 (14.2)	567 (9.4)	1525 (17.5)	
Ethnicity, n (%)				
Hispanic or Latino	2599 (17.6)	762 (12.6)	1837 (21.1)	<0.001
Non Hispanic or Latino	12108 (82.0)	5273 (87.1)	6835 (78.5)	
Unknown	54 (0.4)	17 (0.3)	37 (0.4)	
Pediatric Mortality Risk Scores, median [Q1,Q3]				
Phoenix Score, median [Q1,Q3]	1 [0,2]	1 [0,2]	1 [0,2]	0.037
pSOFA, median [Q1,Q3]	4 [2,6]	4 [2,7]	3 [1,6]	<0.001
PELOD-2, median [Q1,Q3]	4 [2,6]	4 [3,6]	4 [2,5]	<0.001
PRISM III, median [Q1,Q3]	6 [3,11]	7 [3,12]	6 [3,10]	<0.001
Length of Stay (days), median [Q1,Q3]				
Hospital	5.8 [3.4,12.1]	6.5 [3.5,14.0]	5.5 [3.4,10.9]	<0.001
PICU	3.0 [1.8,6.0]	3.0 [1.8,6.3]	2.9 [1.8,5.9]	0.028
Outcomes, n (%)				
Sepsis	4827 (32.7)	2150 (35.5)	2677 (30.7)	<0.001
Septic Shock	2094 (14.2)	988 (16.3)	1106 (12.7)	<0.001
In-Hospital Mortality	491 (3.3)	295 (4.9)	196 (2.3)	<0.001
Composite Outcome^b^	3376 (22.9)	1519 (25.1)	1857 (21.3)	<0.001

*Abbreviations: PELOD-2 - Pediatric Logistic Organ Dysfunction-2, PICU - Pediatric Intensive Care Unit, PRISM III - Pediatric Risk of Mortality III, pSOFA - Pediatric Sequential Organ Assessment. ^a^P-values were computed using the Kruskal-Wallis test. ^b^The composite outcome is defined as having a length of stay in the PICU longer than 7 days or death within 28 days of being in the PICU.

**Fig 2:**
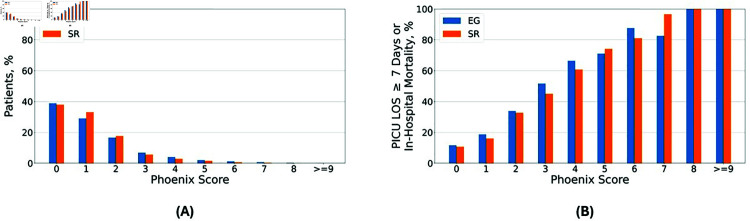
The distribution of the Phoenix Sepsis Score in children with suspected infection and geocodable Georgia residential addresses and composite primary outcome by pediatric intensive care unit (PICU) site. (A) The distribution of children with suspected infection (n = 14,761) by Phoenix Sepsis Score admitted to either the Children’s Healthcare of Atlanta Egleston (EG) PICU (blue bars) or the Scottish Rite (SR) PICU (orange bars). (B) The distribution of the composite primary outcome (in-hospital mortality or persistence of a Phoenix Sepsis Score of  ≥  2 one week following Phoenix sepsis onset) by PICU site.

## Results

### Cohort demographic and clinical characteristics

Out of 63,875 hospitalizations with PICU encounters, there were 14,761 children with geocodable Georgia addressed with suspected infection admitted to the PICU during the study period with 6,052 at EG and 8,709 at SR. The demographic, illness severity scores, and outcome data for children with suspected infection are summarized in Table 1. Children admitted to EG were older (4.3 [1.1–11.4] years vs. 3.4 [0.9–10.0] years, *p* < 0.001) compared those admitted to SR. There was a significantly higher proportion of Black/African American children at EG (50.7%) compared to SR (30.3%), and a significantly higher proportion of Hispanic/Latino children at SR (21.1%) compared to EG (12.6%). The distribution of children within the derivation and validation cohorts by Phoenix Sepsis Score and their relationship with the composite primary outcome is shown in Fig 2. Of the children with suspected infection, there were 2,150 (35.5%) children at EG and 2,677 (30.7%) at SR with a Phoenix Sepsis Score  ≥  2 (*p* < 0.001). Additionally, there were 997 (16.3%) children at EG and 1,114 (12.7%) at SR with Phoenix septic shock (*p* < 0.001). There were 295 (4.9%) children with suspected infection at EG and 196 (2.2%) at SR who died in the hospital (*p* < 0.001). The composite primary outcome occurred in 1,519 (25.1%) at EG and in 1,857 (21.3%) at SR (*p* < 0.001). Measures of center and variability for the vital signs, laboratory tests, and clinical features in the derivation and validation datasets are shown in S2 and S3 Tables. Entry density boxplots indicating the distribution of missingness for the clinical variables in the derivation and validation cohorts used for model development are shown in S2 Fig. Additional information on the change of mortality and comorbidity of the study cohort over the years of admission can be found in S5 Table, and additional information on the differences in the organ failure patterns of the EG and SR PICUs can be found in S6 Table.

### Child opportunity index population differences by hospital site

The population-level Georgia-normed COI 3.0 values for the subdomains, domains, and overall rank by EG and SR sites for children with suspected infection are shown in Table 2. For all COI 3.0 variables, children admitted at the EG site live in lower opportunity neighborhoods compared to children admitted at the SR site. The largest difference in COI 3.0 is within the Education Domain (38 [14–64] vs. 58 [33–79], *p* < 0.001) followed by the Social and Economic Domain (41 [20–64] vs. 57 [32–79], *p* < 0.001) and the Health and Environment Domain (43 [25–63] vs. 53 [28–70], *p* < 0.001). The difference within the Education Domain is driven by the Elementary Education subdomain along with differences in Secondary and Post-secondary Education and Education Resources (Table 2). The difference within the Social and Economic Domain is driven by the Economic and Social Resources subdomains along with differences in Employment, Wealth, and Concentrated Socioeconomic Inequity (Table 2). There is a skew in the EG PICU cohort toward low opportunity neighborhoods where 51% of children admitted to the EG PICU live within the two lowest COI 3.0 quintiles (COI 3.0  ≤  40%) compared to 33% of children admitted to the SR PICU. Conversely, 57% of children admitted to the SR PICU live in the two highest COI 3.0 neighborhoods (COI 3.0 > 60%) compared to 29% of children admitted to the EG PICU. Of children who died in the hospital, 56% versus 33% lived in the two lowest COI 3.0 quintiles at EG and SR, respectively.

**Table 2:** Population-Level Childhood Opportunity Index 3.0 Scores by Domain and Subdomains for Children with Phoenix Sepsis by Location of Pediatric Intensive Care Unit (PICU).

Characteristic, median [Q1,Q3]	Total encounters	EG	SR	p-value^a^
	n = 14761 (100%)	n = 6052 (41%)	n = 8709 (59%)	
**Education Domain - State-normed^b^**	50 [22,74]	38 [14,64]	58 [33,79]	<0.001
Early Childhood Education	57 [30,76]	52 [27,73]	59 [32,79]	<0.001
Elementary Education	48 [23,73]	35 [14,63.2]	56 [32,77]	<0.001
Secondary and Post-secondary Education	46 [20,74]	37 [14,63]	52 [27,80]	<0.001
Education Resources	49 [25,74]	38 [19,63]	56 [32,79]	<0.001
**Health and Environment Domain - State-normed^b^**	48 [27,68]	43 [25,63]	53 [28,70]	<0.001
Pollution	4 [3,5]	4 [3,8]	4 [3,5]	<0.001
Healthy Environments	40 [30,59]	35 [27,51]	43 [32,63]	<0.001
Safety-related Resources	54 [32,74]	49 [28,72]	58 [36,77]	<0.001
Health Resources	49 [24,74]	46 [25,71]	51 [24,77]	<0.001
**Social and Economic Domain - State-normed^b^**	50 [26,73]	41 [20,6]	57 [32,79]	<0.001
Employment	50 [2,74]	43 [23,62]	57 [33,80]	<0.001
Economic Resources	51 [26,74]	41 [19,66]	57 [33,79]	<0.001
Concentrated Socioeconomic Inequity	47 [26,71]	40 [22,62]	52 [29,76]	<0.001
Housing Resources	47 [27,73]	41 [23,64]	52 [29,76]	<0.001
Social Resources	51 [27,75]	43 [21,65]	60 [36,8]	<0.001
Wealth	50 [24,74]	41 [19,67]	55 [29,79]	<0.001
**Overall Percentile Ranking - State-normed** ^b^	50 [26,74]	40 [19,63]	57 [33,79]	<0.001

^a^P-values were computed using the Kruskal-Wallis test. ^b^State-normed values for Georgia were used.

### Model performance

The model performance metrics predicting the composite primary outcome for those children admitted to a PICU with suspected infection at a fixed sensitivity of 85% are reported in Tables 3 and 4. Using the EG PICU as the derivation site, the XGB model had an internal validation AUROC of 0.84 (95% CI: 0.84–0.85) and an external validation AUROC of 0.80 (95% CI: 0.80–0.81) (Table 3). Using the SR PICU as the derivation site, the XGB model had an internal validation AUROC of 0.83 (95% CI: 0.82–0.83) and an external validation AUROC of 0.82 (95% CI: 0.81–0.82) (Table 4). Using the EG PICU as the derivation site, the XGB model had an internal validation AUPRC of 0.68 (95% CI: 0.67–0.69) and an external validation AUPRC of 0.59 (95% CI: 0.58–0.59) (Table 3). Using the SR PICU as the derivation site, the XGB model had an internal validation AUPRC of 0.63 (95% CI: 0.61–0.64) and an external validation AUROC of 0.65 (95% CI: 0.64–0.65) (Table 4). The model performs marginally better when the SR site is used to train the model as compared to when the EG site is used to train the model. There was no improvement in any of the model metrics with the inclusion of the COI 3.0 variables to the model. The AUROC, AUPRC, and calibration plots for the internal and external validation of the EG and SR PICU sites can be seen in S3–S8 Figs. Additionally, the performance results of the models using only COI features are included in S4 Table.

### Important model features

**Fig 3:**
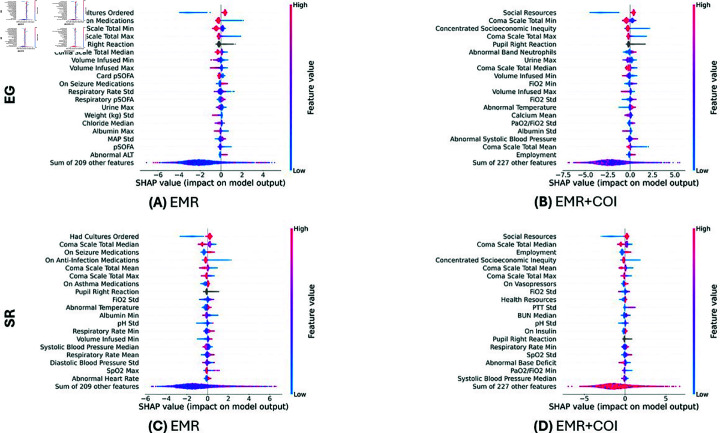
SHAP beeswarm plot of the top 20 features for the eXtreme gradient boosting (XGB) models predicting the composite primary outcome of in-hospital mortality or persistence of a Phoenix sepsis score  ≥  2 one week following Phoenix sepsis onset. The SHAP summary plot explaining the relative importance of features for the models trained with Egleston (EG) data (A) with electronic medical record (EMR) data or with (B) EMR and Child Opportunity Index (COI) data and for the models trained with Scottish Rite (SR) data with (C) EMR data or with (D) EMR and COI data.

The top 20 most important model features contributing to the prediction of the composite primary outcome in children with suspected infection are shown in the SHAP beeswarm plots in Fig 3. For the models trained only on EMR data, the top features in the models included clinical binary indicators, such as whether the patient had cultures ordered, was on any medications for infections, asthma, or seizures, or had any abnormal vital sign measurements or laboratory test results (Fig 3A and 3C). Additionally, features related to pSOFA components and the Glasgow Coma Scale (GCS) total score also appeared in the top features (Fig 3A and 3C). For the models trained with both EMR and COI data, features related to the GCS total score and clinical binary indicators were among the top features (Fig 3B and 3D), similar to what was observed in the models trained only on EMR data. However, COI features related to the social and economic domain, including social resources, concentrated socioeconomic inequity, and employment, also appeared in the top features (Fig 3B and 3D).

**Table 3:** Model performance results using Egleston (EG) as the internal validation site and Scottish Rite (SR) as the external validation site.

Characteristic Mean (95% CI)	EMR	EMR+COI	p-value^a^
Accuracy			
Internal	0.69 (0.67, 0.70)	0.69 (0.67, 0.71)	0.702
External	0.62 (0.62, 0.63)	0.63 (0.62, 0.63)	0.218
F1			
Internal	0.58 (0.57, 0.59)	0.58 (0.57, 0.59)	0.69
External	0.49 (0.49, 0.49)	0.49 (0.49, 0.50)	0.22
PPV (Precision)			
Internal	0.44 (0.43, 0.45)	0.44 (0.43, 0.46)	0.665
External	0.35 (0.34, 0.35)	0.35 (0.35, 0.35)	0.22
Specificity			
Internal	0.63 (0.61, 0.65)	0.64 (0.61, 0.66)	0.692
External	0.56 (0.56, 0.57)	0.57 (0.56, 0.57)	0.218
AUROC			
Internal	0.84 (0.84, 0.85)	0.84 (0.84, 0.85)	0.417
External	0.81 (0.80, 0.81)	0.81 (0.80, 0.81)	0.705
AUPRC			
Internal	0.68 (0.67, 0.69)	0.68 (0.67, 0.69)	0.932
External	0.59 (0.58, 0.59)	0.59 (0.58, 0.59)	0.104

^*^Abbreviations: EMR - Electronic Medical Record, COI - Childhood Opportunity Index. ^*^The probability prediction threshold was set to fix the sensitivity to 85%. ^a^P-values were computed using the t-test.

**Table 4:** Model performance results using Scottish Rite (SR) as the internal validation site and Egleston (EG) as the external validation site.

Characteristic Mean (95% CI)	EMR	EMR+COI	p-value^a^
Accuracy			
Internal	0.66 (0.64, 0.67)	0.66 (0.65, 0.67)	0.224
External	0.65 (0.64, 0.65)	0.64 (0.64, 0.65)	0.66
F1			
Internal	0.51 (0.50, 0.52)	0.52 (0.51, 0.53)	0.256
External	0.55 (0.54, 0.55)	0.54 (0.54, 0.55)	0.638
PPV (Precision)			
Internal	0.37 (0.36, 0.38)	0.37 (0.37, 0.38)	0.272
External	0.40 (0.40, 0.41)	0.40 (0.40, 0.40)	0.646
Specificity			
Internal	0.60 (0.59, 0.62)	0.61 (0.60, 0.62)	0.224
External	0.58 (0.57, 0.58)	0.57 (0.57, 0.58)	0.669
AUROC			
Internal	0.82 (0.82, 0.83)	0.83 (0.82, 0.83)	0.016
External	0.82 (0.81, 0.82)	0.81 (0.81, 0.81)	0.002
AUPRC			
Internal	0.62 (0.61, 0.63)	0.62 (0.62, 0.63)	0.143
External	0.64 (0.64, 0.65)	0.63 (0.63, 0.64)	0.004

^*^Abbreviations: EMR - Electronic Medical Record, COI - Childhood Opportunity Index. ^*^The probability prediction threshold was set to fix the sensitivity to 85%. ^a^P-values were computed using the t-test.

## Discussion

In this study, we demonstrate the similar performance of an XGB model predicting in-hospital mortality or the persistence of a Phoenix sepsis score  ≥  2 one week following Phoenix sepsis onset in children with suspected infection who were admitted to one of two independently staffed PICUs within the Children’s Healthcare of Atlanta system despite the significant differences in baseline SDoH and in-hospital mortality and persistence of Phoenix sepsis between the two sites. Children living in neighborhoods in the two lowest COI 3.0 quintiles were over-represented in the EG PICU while children living in neighborhoods in the two highest COI 3.0 quintiles were over-represented in the SR PICU. Physiologic variables abstracted from the electronic medical record were the dominant features contributing to the composite primary outcome. While the children with suspected infection admitted to the EG PICU had higher median severity of illness metrics such as the pSOFA, Pediatric Logistic Organ Dysfunction-2 (PELOD-2), and Pediatric Risk of Mortality III (PRISM III) scores, the XGB model performed similarly when externally validated on the SR cohort. Moreover, when trained on the SR data, the XGB model performed slightly better when externally validated on the EG cohort.

Our findings are consistent with results from other studies where children living in the lowest COI neighborhoods are disproportionately represented in PICU admissions, emergency department visits, and emergent PICU readmissions 1-year after survival from a critical illness [1,4,10,12,27]. Consistent with our findings, a previous study of PICU admissions using a large administrative database did not show a significant difference in the odds of PICU mortality using the COI 2.0 from 15 U.S. PICUs [1]. The Education and Social and Economic domains as compared to the Health and Environment domain were more disparate between the EG and SR sites consistent with the results seen in a prior publication [1]. In a systematic review of studies in adults evaluating the association between socioeconomics and mortality within 30-days of intensive care unit admission, the random effects pooled analysis showed that lower socioeconomic position was associated with higher mortality with a pooled odds ratio of 1.13 (95% CI: 1.05-1.22) [28]. With the exception of pediatric asthma[9], prior studies have analyzed the relationship between neighborhood COI and all children admitted to a PICU regardless of primary diagnosis. This study is the first to use the new pediatric Phoenix sepsis definition to determine the association of the COI 3.0 with a cohort of critically ill children with suspected infection who either die in the hospital or have persistence of a Phoenix sepsis score  ≥  2 at one-week following sepsis onset. Unlike studies that use administrative data, we are able to use readily available physiologic, laboratory, and medication data from the electronic medical record to determine specific factors beyond composite severity of illness scores that could account for the higher odds of in-hospital death or protracted clinical course.

The most important variables adding value to the predictive model were those related to neurologic and cardiovascular function as represented by features including the GCS, pupil size (a component of the GCS), the neurologic pSOFA score, and lactic acid.

Strengths of this study include a large number of patients from two Atlanta-based PICUs that share an electronic medical record. Addresses were mapped by census tract rather than zip code allowing the more accurate assignment of neighborhood attributes to each patient. The use of the updated state-level COI 3.0 is beneficial as this index is multi-faceted and comprised of specific child-centric indicators of neighborhood SDoH. We calculated multiple severity of illness scores at the time of PICU admission to characterize baseline risk of morbidity and mortality relative to COI.

### Limitations

Our study is limited by the use of retrospective data obtained from an electronic medical record within a single pediatric healthcare system to predict in-hospital mortality or persistence of Phoenix sepsis. Generalizability to other pediatric healthcare systems is a concern yet counteracted by the similar performance of the model despite disparate baseline patient and neighborhood-level characteristics.

We did not capture comorbid conditions or medical complexity in this study which may account for the differences in morbidity and mortality in this study. Due to the ELCS capabilities of the EG PICU, a disproportionate number of children with medical complexity and chronic organ dysfunction may have been cared for at the EG PICU compared to the SR PICU thus accounting, at least partially, for the differences in in-hospital mortality and persistence of Phoenix sepsis. In fact, we found that there were a total of 45 patients who were discharged from the SR PICU and admitted to the EG PICU on the same day, most likely due to the difference in ELCS capabilities. However, the incorporation of the pSOFA components in our study may help to mitigate the lack of comorbidity information.

We did not have access to the blood, urine, respiratory, or cerebrospinal fluid (CSF) culture results for the study cohort. As a result, we cannot comment on the particular pathogens that exist within the cohort.

As this was a retrospective study, the electronic medical records do not capture sociodemographic information about individual patients other than proxies for SDoH such as primary insurance payor, primary language spoke, and unmodifiable demographic information such as race and ethnicity. As a result, using neighborhood-level SDoH metrics may inaccurately attribute group-level sociodemographic features to an individual. This phenomenon is referred to as ecological fallacy and is a common error in public health that occurs when a researcher assumes that a relationship observed at the group level also applies to individuals within that group. This can lead to incorrect conclusions or misguided interventions.

It is likely that changes in clinical practice over the 10-year study period occurred; however, the influence of these practice changes cannot be accounted for in a retrospective study design.

Finally, we did not assess longer-term mortality or morbidity including readmission to a PICU at some point within the year following index admission to the hospital – an outcome that may be more sensitive to neighborhood-level SDoH exposures.

## Conclusion

The SDoH are fundamental factors that contribute to an individual’s health and their risk of bad outcomes such as critical illness. In this work, we investigate whether differences in characteristics from the COI 3.0, a child neighborhood environment indicator developed from the SDoH, have a strong association with poor outcomes such as mortality or persistence of organ dysfunction for a week or longer after the onset of Phoenix sepsis. Despite significant differences in COI 3.0 characteristics and in-hospital mortality rates between the two PICUs, the addition of COI 3.0 variables for model development did not result in significant improvement in predictive performance. For future research, further analysis of the COI 3.0 features against different outcomes, such as the risk of readmission to the PICU, must be conducted.

## Supporting information

S1 Fig Data preprocessing pipeline SHAP = Shapley values indicating features of importance in the model
(TIF)

S2 Fig Entry density boxplots indicate the distribution of missingness for the clinical variables in the (A) derivation and (B) validation cohorts used in the models (TIF)

S3 Fig The area under the receiver operating characteristic (AUROC) curve for the Egleston campus predicting in-hospital mortality in children with suspected infection under the Phoenix criteria for the (A) internal or (B) external validation dataset for the eXtreme Gradient Boosting (XGB) models trained on electronic medical records (EMR), EMR and Child Opportunity Index (COI) indicators (EMR+COI), and COI indicators respectively Recall (sensitivity) was fixed at 0.85 and denoted by a + sign
(TIF)

S4 Fig The area under the precision-recall curve (AUPRC) for the Egleston campus predicting in-hospital mortality in children with suspected infection under the Phoenix criteria for the (A) internal or (B) external validation dataset for the eXtreme Gradient Boosting (XGB) models trained on electronic medical records (EMR), EMR and Child Opportunity Index (COI) indicators (EMR+COI), and COI indicators respectively Recall (sensitivity) was fixed at 0.85 and denoted by a + sign
(TIF)

S5 Fig Calibration plots for the Egleston campus predicting in-hospital mortality in children with suspected infection under the Phoenix criteria for the (A) internal or (B) external validation dataset for the eXtreme Gradient Boosting (XGB) models trained on electronic medical records (EMR), EMR and Child Opportunity Index (COI) indicators (EMR+COI), and COI indicators respectively (TIF)

S6 Fig The area under the receiver operating characteristic (AUROC) curve for the Scottish Rite campus predicting in-hospital mortality in children with suspected infection under the Phoenix criteria for the (A) internal or (B) external validation dataset for the eXtreme Gradient Boosting (XGB) models trained on electronic medical records (EMR), EMR and Child Opportunity Index (COI) indicators (EMR+COI), and COI indicators respectively Recall (sensitivity) was fixed at 0.85 and denoted by a + sign
(TIF)

S7 Fig The area under the precision-recall curve (AUPRC) for the Scottish Rite campus predicting in-hospital mortality in children with suspected infection under the Phoenix criteria for the (A) internal or (B) external validation dataset for the eXtreme Gradient Boosting (XGB) models trained on electronic medical records (EMR), EMR and Child Opportunity Index (COI) indicators (EMR+COI), and COI indicators respectively Recall (sensitivity) was fixed at 0.85 and denoted by a + sign
(TIF)

S8 Fig Calibration plots for the Scottish Rite campus predicting in-hospital mortality in children with suspected infection under the Phoenix criteria for the (A) internal or (B) external validation dataset for the eXtreme Gradient Boosting (XGB) models trained on electronic medical records (EMR), EMR and Child Opportunity Index (COI) indicators (EMR+COI), and COI indicators respectively (TIF)

S1 Table List of the vital sign, laboratory test, demographic, and clinical features from the electronic medical record that were used in the model (DOCX)

S2 Table Measures of center and variability for vital signs, laboratory tests, and clinical features for those children who met the Phoenix Sepsis Criteria in the Egleston campus (DOCX)

S3 Table Measures of center and variability for vital signs, laboratory tests, and clinical features for those children who met the Phoenix Sepsis Criteria in the Scottish Rite campus (DOCX)

S4 Table Model performance results using the Child Opportunity Index (COI) indicators only for the Egleston and Scottish Rite campuses (DOCX)

S5 Table Annual mortality and comorbidity statistics of the study cohort by site (DOCX)

S6 Table Phoenix and pediatric sequential organ failure assessment (pSOFA) subscore characteristics of the study cohort by site (DOCX)

S7 Table Characteristics of the excluded cohort by site (DOCX)
