# Supplementary figures and images for: Association of the child opportunity index with in-hospital mortality and persistence of organ dysfunction at one week after onset of Phoenix Sepsis among children admitted to the pediatric intensive care unit with suspected infection
Source: PLOS Digit Health. 2025 Apr 14;4(4):e0000763. doi: 10.1371/journal.pdig.0000763 (PMC11996216; doi:10.1371/journal.pdig.0000763)

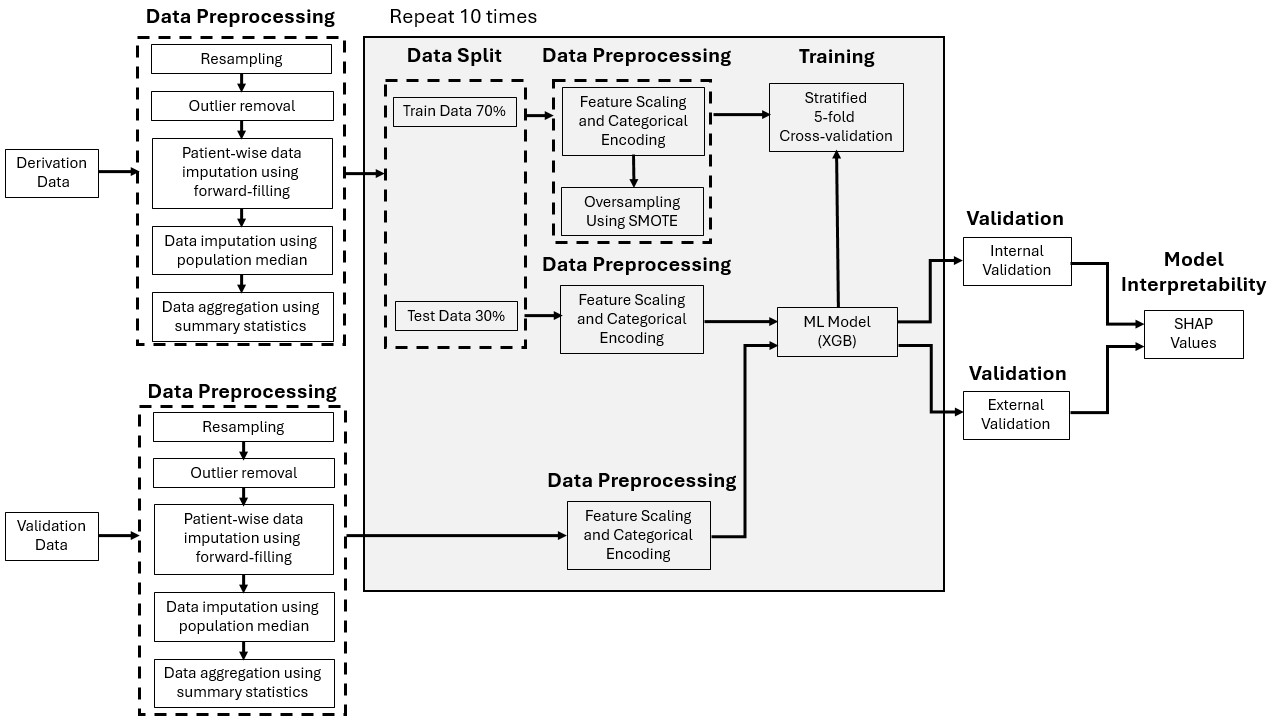

Supplement: S1 Fig — SHAP = Shapley values indicating features of importance in the model (TIF) [file pdig.0000763.s001.tif]

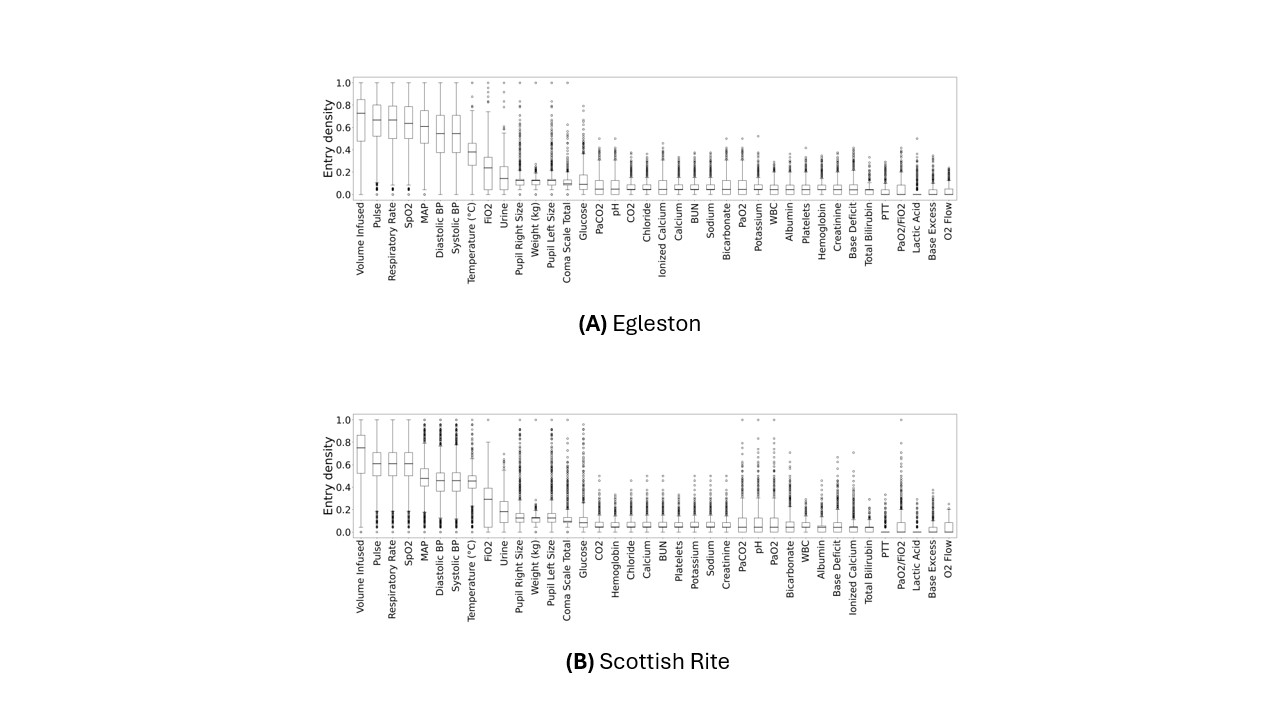

Supplement: S2 Fig — (TIF) [file pdig.0000763.s002.tif]

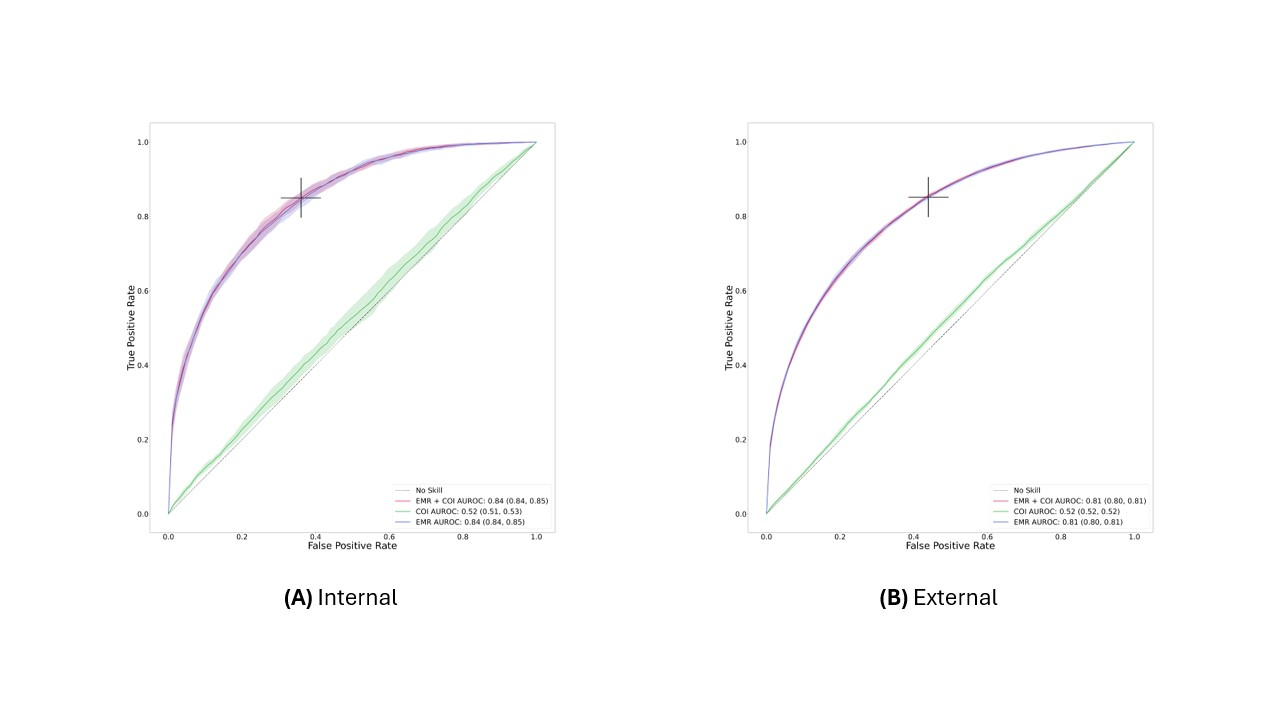

Supplement: S3 Fig — Recall (sensitivity) was fixed at 0.85 and denoted by a + sign (TIF) [file pdig.0000763.s003.tif]

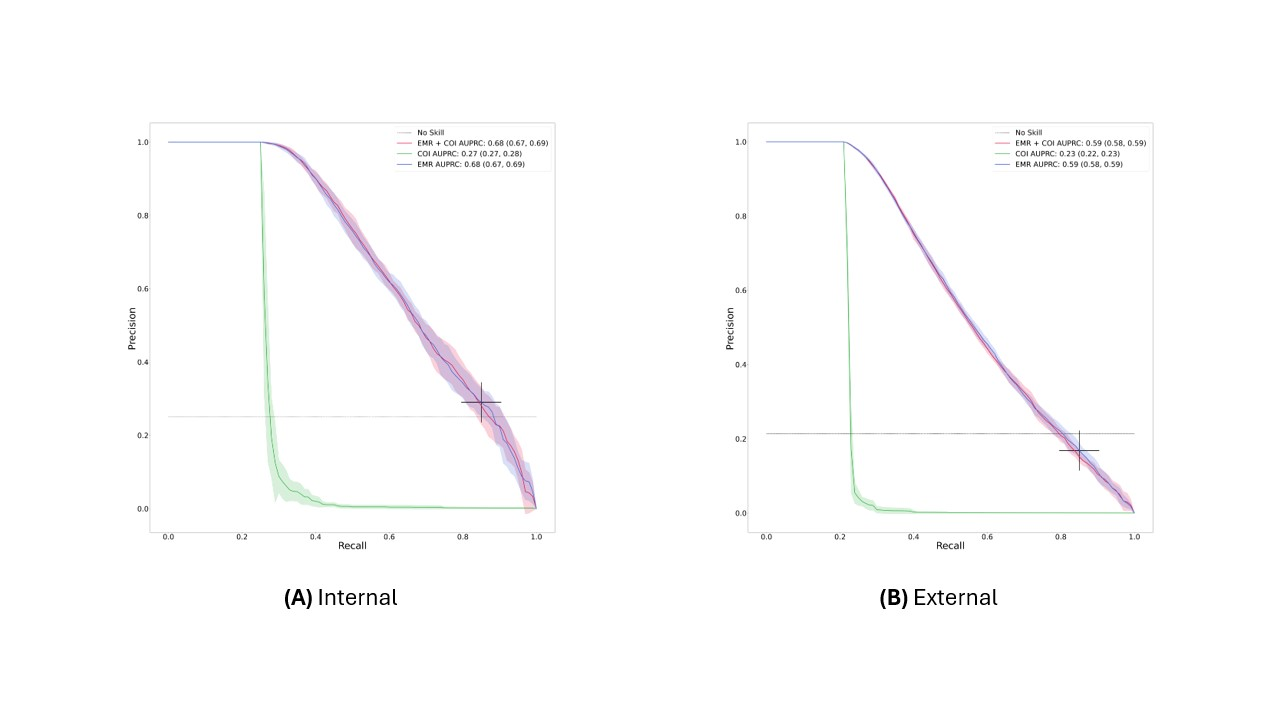

Supplement: S4 Fig — Recall (sensitivity) was fixed at 0.85 and denoted by a + sign (TIF) [file pdig.0000763.s004.tif]

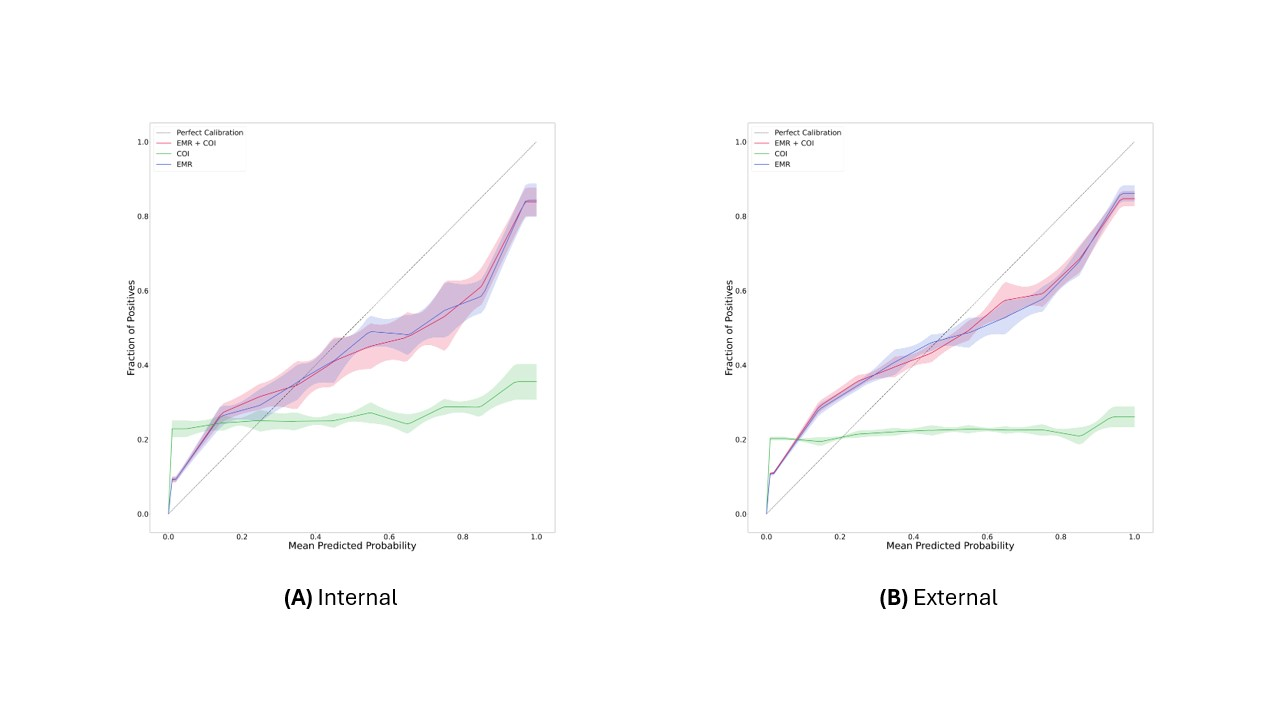

Supplement: S5 Fig — (TIF) [file pdig.0000763.s005.tif]

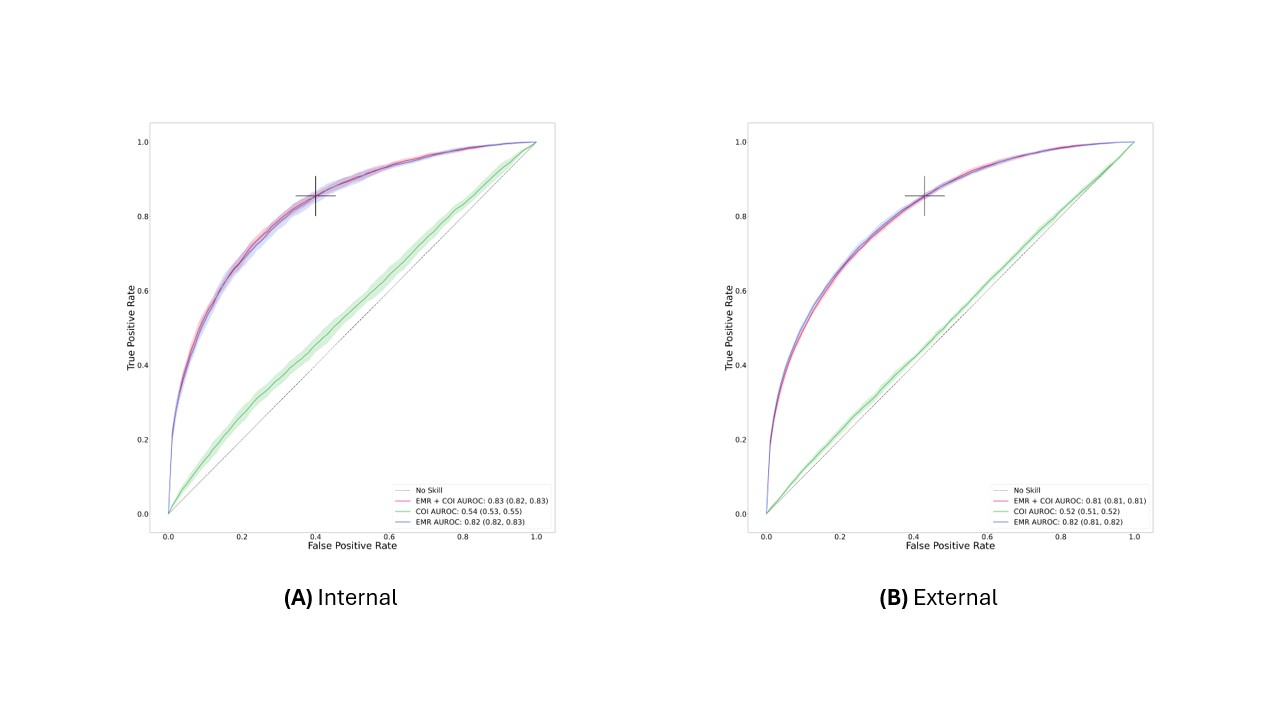

Supplement: S6 Fig — Recall (sensitivity) was fixed at 0.85 and denoted by a + sign (TIF) [file pdig.0000763.s006.tif]

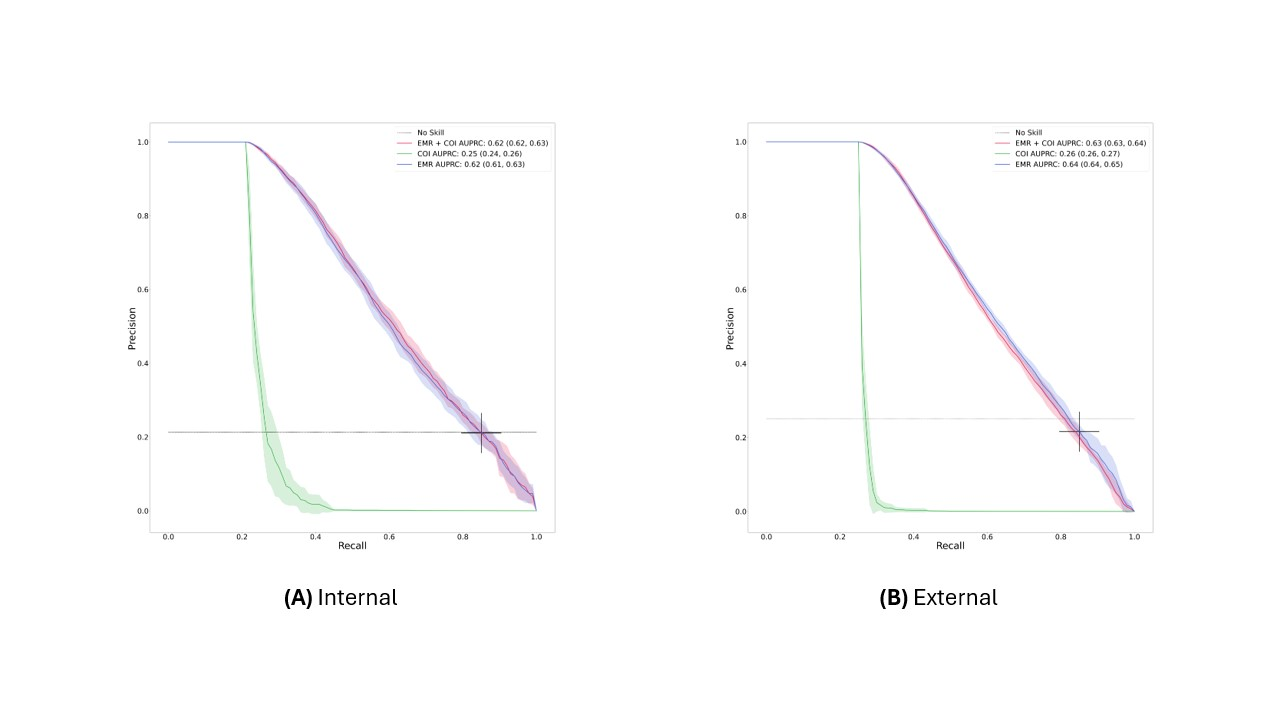

Supplement: S7 Fig — Recall (sensitivity) was fixed at 0.85 and denoted by a + sign (TIF) [file pdig.0000763.s007.tif]

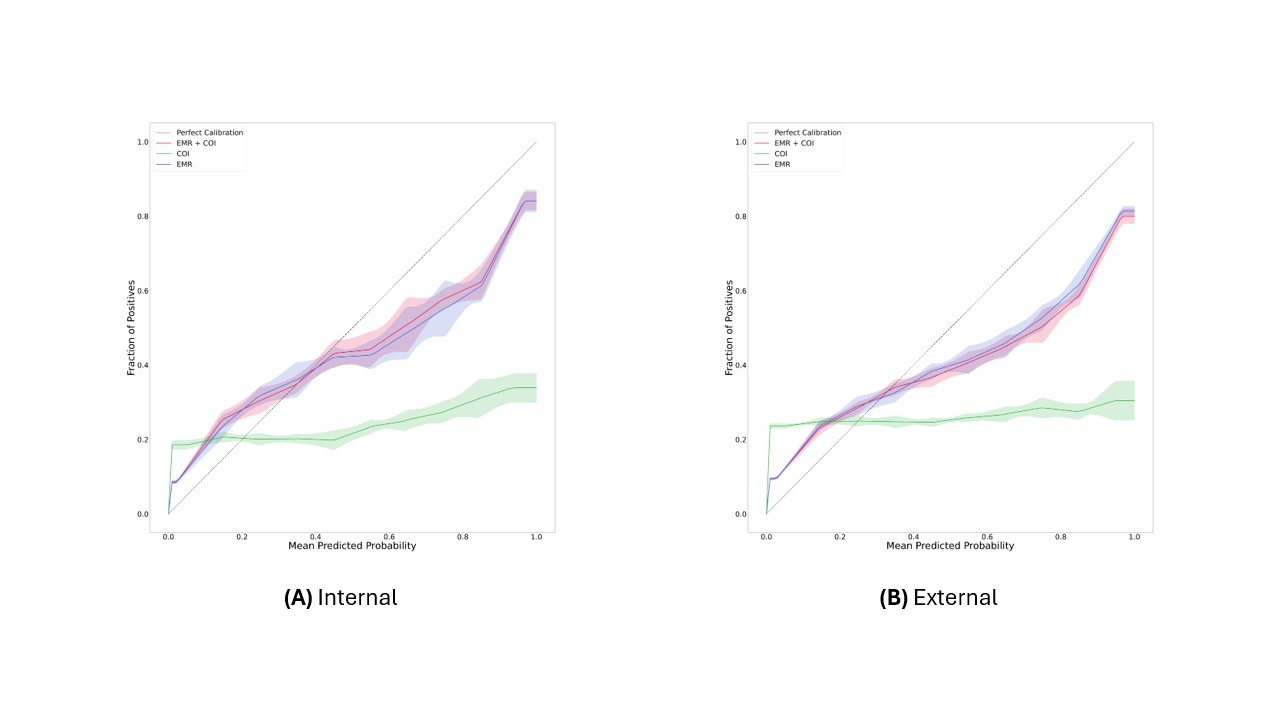

Supplement: S8 Fig — (TIF) [file pdig.0000763.s008.tif]
