# Supplementary material for: Association of the child opportunity index with in-hospital mortality and persistence of organ dysfunction at one week after onset of Phoenix Sepsis among children admitted to the pediatric intensive care unit with suspected infection
Source: PLOS Digit Health. 2025 Apr 14;4(4):e0000763. doi: 10.1371/journal.pdig.0000763 (PMC11996216; doi:10.1371/journal.pdig.0000763)
Supplement: S1 Table — (DOCX) [file pdig.0000763.s009.docx]

**S1 Table.** List of the vital sign, laboratory test, demographic, and clinical features from the electronic medical record that were used in the model.

| **Feature Type** | **Feature(s)** |
| --- | --- |
| Demographics | Age (years), Age (months), Weight (kg) |
| Vital Signs | DBP, SBP, Temperature (Celsius), GCS Total, MAP, SpO2, Respiratory Rate, Pulse |
| Laboratory Values | Albumin, Base Deficit, Base Excess, Bicarbonate, Total Bilirubin, BUN, Calcium, Ionized Calcium, Chloride, CO2, Creatinine, FiO2, Glucose, Hemoglobin, Lactic Acid, O2 Flow, PaO2/FiO2, PaCO2, pH, Platelets, PaO2, Potassium, PTT, Sodium, WBC |
| Clinical Variables | Urine, Volume Infused, Left Pupil Reaction, Right Pupil Reaction, Left Pupil Size, Right Pupil Size, pSOFA, Coagulation pSOFA, Respiratory pSOFA, Hepatic pSOFA, Cardiovascular pSOFA, Neurologic pSOFA, Renal pSOFA, Abnormal Heart Rate, Abnormal Respiratory Rate, Abnormal Temperature, Abnormal WBC, Abnormal Band Neutrophils, Abnormal SBP, Abnormal Base Deficit, Abnormal Lactate, Constant SpO2 Below 90, FiO2 Above 50, Low Platelets, Abnormal Prothrombin Time, Abnormal INR, Elevated Creatinine, Abnormal ALT, Abnormal AST, On Asthma Medications, On Seizure Medications, On Vasopressors, On Anti-Infection Medications, On Insulin, Had Cultures Ordered, Sepsis Septicemia Diagnosis, Septic Shock Diagnosis, Sickle Cell Diagnosis, DKA Diagnosis, Asthmaticus Diagnosis |

Abbreviations: DBP – Diastolic Blood Pressure, SBP – Systolic Blood Pressure, GCS – Glasgow Coma Scale, MAP – Mean Arterial Pressure, SpO2 – Pulse Oximetry, BUN – Blood Urea Nitrogen, CO2 – Carbon Dioxide, FiO2 – Fraction of Inspired Oxygen, O2 – Oxygen, PaO2 – Partial Pressure of Oxygen, PaCO2 – Partial Pressure of Carbon Dioxide, PTT – Partial Thromboplastin Time, WBC – White Blood Cell Count, pSOFA – Pediatric Sequential Organ Failure Assessment, INR – International Normalized Ratio, ALT – Alanine Aminotransferase, AST – Aspartate Aminotransferase, DKA – Diabetic Ketoacidosis.
