# Supplementary material for: Association of the child opportunity index with in-hospital mortality and persistence of organ dysfunction at one week after onset of Phoenix Sepsis among children admitted to the pediatric intensive care unit with suspected infection
Source: PLOS Digit Health. 2025 Apr 14;4(4):e0000763. doi: 10.1371/journal.pdig.0000763 (PMC11996216; doi:10.1371/journal.pdig.0000763)
Supplement: S2 Table — (DOCX) [file pdig.0000763.s010.docx]

**S2 Table.** Measures of center and variability for vital signs, laboratory tests, and clinical features for those children who met the Phoenix Sepsis Criteria in the Egleston campus.

|  | **Egleston** | | | | | | |
| --- | --- | --- | --- | --- | --- | --- | --- |
| **Feature** | **Min** | **Max** | **Mean** | **Median** | **Q1** | **Q3** | **Std** |
| Albumin | 1.4 | 4.9 | 2.9 | 2.9 | 2.4 | 3.3 | 0.6 |
| Base Deficit | 1.0 | 26.0 | 5.7 | 5.0 | 3.0 | 7.0 | 4.4 |
| Base Excess | 0.0 | 18.0 | 2.9 | 2.0 | 0.0 | 4.0 | 3.4 |
| Bicarbonate | 4.8 | 38.5 | 22.4 | 22.4 | 19.6 | 25.3 | 5.2 |
| Total Bilirubin | 0.1 | 15.5 | 1.1 | 0.4 | 0.3 | 0.9 | 2.0 |
| Diastolic Blood Pressure | 30.0 | 98.0 | 59.9 | 59.0 | 50.0 | 69.0 | 14.0 |
| Systolic Blood Pressure | 65.0 | 147.0 | 103.0 | 103.0 | 92.0 | 114.0 | 16.1 |
| Blood Urea Nitrogen | 2.0 | 81.0 | 15.2 | 11.0 | 7.0 | 17.0 | 13.0 |
| Calcium | 6.1 | 10.6 | 8.4 | 8.4 | 7.8 | 8.9 | 0.8 |
| Ionized Calcium | 2.6 | 6.3 | 4.7 | 4.8 | 4.4 | 5.1 | 0.6 |
| Chloride | 90.0 | 136.0 | 109.9 | 109.0 | 105.0 | 114.0 | 7.8 |
| CO2 | 5.0 | 34.0 | 21.4 | 22.0 | 19.0 | 24.0 | 4.8 |
| GCS Total | 3.0 | 15.0 | 11.5 | 14.0 | 8.0 | 15.0 | 4.1 |
| Creatinine | 0.2 | 7.7 | 0.7 | 0.5 | 0.3 | 0.8 | 1.0 |
| FiO2 | 21.0 | 100.0 | 48.4 | 40.0 | 30.0 | 60.0 | 26.2 |
| Glucose | 56.5 | 582.0 | 137.9 | 117.0 | 95.0 | 154.0 | 68.4 |
| Hemoglobin | 4.8 | 17.5 | 10.8 | 10.9 | 9.3 | 12.3 | 2.2 |
| Lactic Acid | 0.4 | 23.4 | 3.5 | 2.2 | 1.2 | 4.4 | 3.5 |
| MAP | 42.0 | 109.0 | 72.3 | 71.5 | 63.0 | 81.0 | 13.4 |
| O2 Flow | 0.0 | 2.6 | 0.5 | 0.3 | 0.1 | 0.8 | 0.5 |
| PaO2/FiO2 | 21.0 | 1623.8 | 194.3 | 152.5 | 93.8 | 250.0 | 148.8 |
| PCO2 | 17.0 | 85.8 | 43.2 | 41.2 | 35.1 | 49.2 | 11.8 |
| pH | 7.0 | 7.5 | 7.3 | 7.3 | 7.3 | 7.4 | 0.1 |
| Platelets | 23.0 | 753.0 | 255.5 | 239.0 | 157.0 | 336.0 | 136.6 |
| PO2 | 21.0 | 349.0 | 77.2 | 56.5 | 41.0 | 90.0 | 56.2 |
| Potassium | 2.4 | 6.9 | 4.0 | 3.9 | 3.5 | 4.5 | 0.8 |
| PTT | 21.3 | 129.3 | 37.5 | 33.5 | 29.3 | 40.4 | 14.3 |
| Pulse | 58.0 | 190.0 | 125.1 | 126.0 | 105.0 | 145.5 | 27.9 |
| Pupil Left Size | 1.0 | 6.0 | 2.8 | 3.0 | 2.0 | 3.0 | 1.0 |
| Pupil Right Size | 1.0 | 6.0 | 2.8 | 3.0 | 2.0 | 3.0 | 0.9 |
| Respiratory Rate | 11.0 | 66.0 | 28.6 | 26.0 | 20.0 | 35.0 | 11.4 |
| Sodium | 126.0 | 168.0 | 141.4 | 140.0 | 137.0 | 144.0 | 7.0 |
| SpO2 | 79.0 | 100.0 | 97.6 | 99.0 | 96.5 | 100.0 | 3.3 |
| Temperature | 35.2 | 39.3 | 37.0 | 36.9 | 36.5 | 37.4 | 0.7 |
| Urine | 0.0 | 800.0 | 152.5 | 100.0 | 42.0 | 205.0 | 153.6 |
| Volume Infused | 1.0 | 200.0 | 49.1 | 45.0 | 24.0 | 70.0 | 34.2 |
| WBC | 0.9 | 47.0 | 12.3 | 10.8 | 7.1 | 15.8 | 7.4 |
| Weight (kg) | 2.6 | 108.0 | 25.2 | 15.7 | 9.1 | 36.0 | 22.5 |

Abbreviations: DBP – Diastolic Blood Pressure, SBP – Systolic Blood Pressure, BUN – Blood Urea Nitrogen, CO2 – Carbon Dioxide, GCS – Glasgow Coma Scale, FiO2 – Fraction of Inspired Oxygen, MAP – Mean Arterial Pressure, O2 – Oxygen, PaO2 – Partial Pressure of Oxygen, PaCO2 – Partial Pressure of Carbon Dioxide, PTT – Partial Thromboplastin Time, SpO2 – Pulse Oximetry, WBC – White Blood Cell Count.
