# Supplementary material for: Association of the child opportunity index with in-hospital mortality and persistence of organ dysfunction at one week after onset of Phoenix Sepsis among children admitted to the pediatric intensive care unit with suspected infection
Source: PLOS Digit Health. 2025 Apr 14;4(4):e0000763. doi: 10.1371/journal.pdig.0000763 (PMC11996216; doi:10.1371/journal.pdig.0000763)
Supplement: S3 Table — (DOCX) [file pdig.0000763.s011.docx]

**S3 Table.** Measures of center and variability for vital signs, laboratory tests, and clinical features for those children who met the Phoenix Sepsis Criteria in the Scottish Rite campus.

|  | **Scottish Rite** | | | | | | |
| --- | --- | --- | --- | --- | --- | --- | --- |
| **Feature** | **Min** | **Max** | **Mean** | **Median** | **Q1** | **Q3** | **Std** |
| Albumin | 1.4 | 4.9 | 2.9 | 3.0 | 2.5 | 3.4 | 0.6 |
| Base Deficit | 1.0 | 26.0 | 5.1 | 4.0 | 2.0 | 7.0 | 4.1 |
| Base Excess | 0.0 | 18.0 | 2.5 | 2.0 | 0.0 | 3.0 | 2.9 |
| Bicarbonate | 4.8 | 38.5 | 22.6 | 22.6 | 20.0 | 25.4 | 4.8 |
| Total Bilirubin | 0.1 | 15.5 | 0.9 | 0.3 | 0.2 | 0.6 | 1.9 |
| DBP | 30.0 | 98.0 | 60.2 | 59.0 | 50.0 | 70.0 | 14.1 |
| SBP | 65.0 | 147.0 | 102.9 | 103.0 | 93.0 | 113.0 | 14.8 |
| BUN | 2.0 | 81.0 | 12.2 | 9.0 | 6.0 | 14.0 | 10.5 |
| Calcium | 6.1 | 10.6 | 8.6 | 8.6 | 8.1 | 9.1 | 0.8 |
| Ionized Calcium | 2.6 | 6.3 | 4.7 | 4.8 | 4.4 | 5.1 | 0.7 |
| Chloride | 90.0 | 136.0 | 109.5 | 109.0 | 105.0 | 113.0 | 6.9 |
| CO2 | 5.0 | 34.0 | 21.4 | 22.0 | 19.0 | 24.0 | 4.2 |
| GCS Total | 3.0 | 15.0 | 11.9 | 14.0 | 9.0 | 15.0 | 3.9 |
| Creatinine | 0.2 | 7.6 | 0.5 | 0.4 | 0.3 | 0.6 | 0.5 |
| FiO2 | 21.0 | 100.0 | 43.0 | 35.0 | 30.0 | 50.0 | 22.2 |
| Glucose | 56.5 | 579.5 | 132.1 | 114.0 | 95.0 | 146.0 | 61.8 |
| Hemoglobin | 4.8 | 17.5 | 11.4 | 11.4 | 10.1 | 12.7 | 2.1 |
| Lactic Acid | 0.4 | 25.9 | 3.9 | 2.5 | 1.4 | 5.0 | 3.7 |
| MAP | 42.0 | 109.0 | 72.7 | 72.0 | 63.0 | 82.0 | 13.3 |
| O2 Flow | 0.0 | 2.6 | 0.7 | 0.6 | 0.2 | 1.1 | 0.6 |
| PaO2/FiO2 | 21.0 | 1628.6 | 196.6 | 170.0 | 110.0 | 248.6 | 124.8 |
| PCO2 | 17.0 | 85.8 | 42.1 | 40.0 | 34.4 | 47.9 | 11.3 |
| pH | 7.0 | 7.5 | 7.3 | 7.4 | 7.3 | 7.4 | 0.1 |
| Platelets | 23.0 | 752.0 | 288.7 | 273.0 | 195.0 | 365.0 | 134.6 |
| PO2 | 21.0 | 350.0 | 78.2 | 62.0 | 46.0 | 87.0 | 52.7 |
| Potassium | 2.4 | 6.9 | 4.1 | 4.0 | 3.6 | 4.5 | 0.8 |
| PTT | 21.2 | 127.9 | 36.5 | 33.7 | 29.3 | 39.9 | 11.8 |
| Pulse | 58.0 | 190.0 | 126.0 | 127.0 | 106.0 | 147.0 | 28.2 |
| Pupil Left Size | 1.0 | 6.0 | 2.8 | 3.0 | 2.0 | 3.0 | 0.9 |
| Pupil Right Size | 1.0 | 6.0 | 2.8 | 3.0 | 2.0 | 3.0 | 0.9 |
| Respiratory Rate | 11.0 | 66.0 | 29.6 | 28.0 | 21.0 | 36.0 | 11.0 |
| Sodium | 126.0 | 168.0 | 140.5 | 139.0 | 137.0 | 143.0 | 6.1 |
| SpO2 | 79.0 | 100.0 | 97.6 | 98.0 | 96.0 | 100.0 | 2.6 |
| Temperature (°C) | 35.2 | 39.3 | 37.1 | 37.0 | 36.6 | 37.4 | 0.7 |
| Urine | 0.0 | 800.0 | 146.5 | 97.0 | 44.0 | 200.0 | 145.1 |
| Volume Infused | 1.0 | 200.0 | 51.4 | 46.0 | 30.0 | 70.0 | 32.3 |
| WBC | 0.9 | 46.7 | 11.9 | 10.6 | 7.3 | 15.0 | 6.5 |
| Weight (kg) | 2.6 | 108.0 | 22.7 | 14.3 | 8.8 | 30.4 | 20.5 |

Abbreviations: DBP – Diastolic Blood Pressure, SBP – Systolic Blood Pressure, BUN – Blood Urea Nitrogen, CO2 – Carbon Dioxide, GCS – Glasgow Coma Scale, FiO2 – Fraction of Inspired Oxygen, MAP – Mean Arterial Pressure, O2 – Oxygen, PaO2 – Partial Pressure of Oxygen, PaCO2 – Partial Pressure of Carbon Dioxide, PTT – Partial Thromboplastin Time, SpO2 – Pulse Oximetry, WBC – White Blood Cell Count.
