# Supplementary material for: Association of the child opportunity index with in-hospital mortality and persistence of organ dysfunction at one week after onset of Phoenix Sepsis among children admitted to the pediatric intensive care unit with suspected infection
Source: PLOS Digit Health. 2025 Apr 14;4(4):e0000763. doi: 10.1371/journal.pdig.0000763 (PMC11996216; doi:10.1371/journal.pdig.0000763)
Supplement: S4 Table — (DOCX) [file pdig.0000763.s012.docx]

**S4 Table.** Model performance results using the Childhood Opportunity Index (COI) indicators only for the Egleston and Scottish Rite campuses.

| **Characteristic Mean (95% CI)** | **Egleston** | **Scottish Rite** |
| --- | --- | --- |
| Accuracy  Internal  External | 0.34 (0.34, 0.35)  0.30 (0.30, 0.31) | 0.32 (0.31, 0.33)  0.33 (0.33, 0.34) |
| F1  Internal  External | 0.39 (0.39, 0.40)  0.34 (0.34, 0.34) | 0.35 (0.35, 0.35)  0.39 (0.39, 0.39) |
| PPV (Precision)  Internal  External | 0.26 (0.25, 0.26)  0.21 (0.21, 0.22) | 0.22 (0.22, 0.22)  0.25 (0.25, 0.25) |
| Specificity  Internal  External | 0.17 (0.16, 0.18)  0.16 (0.15, 0.16) | 0.18 (0.17, 0.19)  0.16 (0.16, 0.16) |
| AUROC  Internal  External | 0.52 (0.51, 0.53)  0.52 (0.52, 0.52) | 0.54 (0.53, 0.55)  0.52 (0.51, 0.52) |
| AUPRC  Internal  External | 0.27 (0.27, 0.28)  0.23 (0.22, 0.23) | 0.25 (0.24, 0.26)  0.26 (0.26, 0.27) |

Abbreviations: CI – Confidence Interval.

*The probability prediction threshold was set to fix the sensitivity to 85%.
