# Supplementary material for: Association of the child opportunity index with in-hospital mortality and persistence of organ dysfunction at one week after onset of Phoenix Sepsis among children admitted to the pediatric intensive care unit with suspected infection
Source: PLOS Digit Health. 2025 Apr 14;4(4):e0000763. doi: 10.1371/journal.pdig.0000763 (PMC11996216; doi:10.1371/journal.pdig.0000763)
Supplement: S5 Table — (DOCX) [file pdig.0000763.s013.docx]

**S5 Table.** Annual mortality and comorbidity statistics of the study cohort by site.

| **Year** | **2016** | | **2017** | | **2018** | | **2019** | | **2020** | | **2021** | | **2022** | |
| --- | --- | --- | --- | --- | --- | --- | --- | --- | --- | --- | --- | --- | --- | --- |
| **Site** | EG | SR | EG | SR | EG | SR | EG | SR | EG | SR | EG | SR | EG | SR |
| Mortality, n | 60 | 27 | 49 | 34 | 41 | 34 | 48 | 35 | 45 | 25 | 45 | 30 | 7 | 11 |
| **Comorbidity, n** | | | | | | | | | | | | | | |
| Myocardial Infarction | 1 | 0 | 0 | 0 | 0 | 0 | 0 | 0 | 0 | 0 | 0 | 0 | 0 | 0 |
| Congestive Heart Failure | 1 | 0 | 2 | 0 | 0 | 0 | 0 | 0 | 2 | 1 | 1 | 0 | 0 | 0 |
| Peripheral Vascular Disease | 0 | 0 | 1 | 0 | 0 | 0 | 1 | 1 | 0 | 1 | 0 | 3 | 0 | 0 |
| Cerebrovascular  Disease | 6 | 4 | 5 | 2 | 2 | 6 | 10 | 2 | 3 | 1 | 6 | 6 | 3 | 1 |
| Chronic Pulmonary Disease and Pneumonia | 11 | 26 | 11 | 24 | 10 | 33 | 12 | 21 | 4 | 10 | 3 | 13 | 4 | 3 |
| Rheumatic Disease | 1 | 0 | 0 | 0 | 0 | 0 | 1 | 0 | 2 | 8 | 8 | 17 | 0 | 3 |
| Peptic Ulcer Disease | 1 | 1 | 0 | 0 | 0 | 1 | 0 | 0 | 0 | 1 | 1 | 1 | 0 | 0 |
| Mild Liver Disease | 5 | 1 | 3 | 0 | 4 | 0 | 4 | 0 | 7 | 1 | 8 | 0 | 4 | 0 |
| Diabetes | 2 | 0 | 4 | 1 | 3 | 2 | 1 | 2 | 0 | 1 | 2 | 2 | 0 | 1 |
| Hemiplegia or paraplegia | 1 | 1 | 1 | 6 | 1 | 4 | 0 | 7 | 1 | 3 | 3 | 1 | 0 | 0 |
| Renal Disease | 0 | 1 | 0 | 0 | 0 | 0 | 0 | 0 | 1 | 1 | 1 | 0 | 0 | 0 |
| Malignancy | 0 | 3 | 3 | 6 | 5 | 6 | 2 | 4 | 9 | 11 | 8 | 4 | 1 | 1 |
| Moderate or Severe Liver Disease | 1 | 1 | 2 | 1 | 4 | 1 | 0 | 0 | 2 | 1 | 2 | 1 | 1 | 0 |
| Metastatic Solid Tumor | 0 | 0 | 0 | 0 | 0 | 0 | 0 | 0 | 1 | 1 | 0 | 0 | 0 | 0 |

Abbreviations: EG – Egleston, SR – Scottish Rite.
