# Supplementary material for: Association of the child opportunity index with in-hospital mortality and persistence of organ dysfunction at one week after onset of Phoenix Sepsis among children admitted to the pediatric intensive care unit with suspected infection
Source: PLOS Digit Health. 2025 Apr 14;4(4):e0000763. doi: 10.1371/journal.pdig.0000763 (PMC11996216; doi:10.1371/journal.pdig.0000763)
Supplement: S6 Table — (DOCX) [file pdig.0000763.s014.docx]

**S6 Table.** Phoenix and pediatric sequential organ failure assessment (pSOFA) subscore characteristics of the study cohort by site.

| **Characteristic median [Q1, Q3]** | **Total**  **n = 4827**  **(100%)** | **Egleston**  **n = 2150**  **(45%)** | **Scottish Rite**  **n = 2677**  **(55%)** | **p-value*^a^*** |
| --- | --- | --- | --- | --- |
| Phoenix | | | | |
| Respiratory | 1 [0,1] | 1 [0,1] | 1 [0,1] | <0.001 |
| Cardiovascular | 0 [0,1] | 0 [0,1] | 0 [0,1] | 0.129 |
| Coagulation | 0 [0,1] | 1 [0,1] | 0 [0,1] | <0.001 |
| Neurologic | 1 [0,1] | 1 [0,1] | 1 [0,1] | <0.001 |
| pSOFA | | | | |
| Respiratory | 3 [0,4] | 3 [0,4] | 3 [1,4] | 0.036 |
| Coagulation | 0 [0,1] | 0 [0,1] | 0 [0,0] | <0.001 |
| Hepatic | 0 [0,0] | 0 [0,0] | 0 [0,0] | <0.001 |
| Cardiovascular | 1 [0,1] | 1 [0,1] | 1 [1,1] | 0.054 |
| Neurologic | 3 [1,4] | 3 [1,4] | 3 [2,4] | 0.696 |
| Renal | 0 [0,1] | 0 [0,1] | 0 [0,0] | <0.001 |

*a* – P-values were computed using the Kruskal-Wallis test.
