# Supplementary material for: Association of the child opportunity index with in-hospital mortality and persistence of organ dysfunction at one week after onset of Phoenix Sepsis among children admitted to the pediatric intensive care unit with suspected infection
Source: PLOS Digit Health. 2025 Apr 14;4(4):e0000763. doi: 10.1371/journal.pdig.0000763 (PMC11996216; doi:10.1371/journal.pdig.0000763)
Supplement: S7 Table — (DOCX) [file pdig.0000763.s015.docx]

**S7 Table.** Characteristics of the excluded cohort by site.

| **Characteristic median [Q1, Q3] or n (%)** | **Total**  **n = 1987**  **(100%)** | **Egleston**  **n = 797**  **(41%)** | **Scottish Rite**  **n = 1190**  **(59%)** | **p-value*^a^*** |
| --- | --- | --- | --- | --- |
| Age, years | 5.7 [1.7,12.9] | 5.5 [1.7,11.9] | 5.9 [1.8,13.7] | 0.087 |
| Age Group | | | | |
| ≤ 28 days | 78 (3.9) | 29 (3.6) | 49 (4.1) | 0.698 |
| 29 days - 2 years | 651 (32.8) | 272 (34.1) | 379 (31.8) |  |
| 3 - 5 years | 292 (14.7) | 118 (14.8) | 174 (14.6) |  |
| 6 - 17 years | 966 (48.6) | 378 (47.4) | 588 (49.4) |  |
| Sex | | | | |
| Female | 892 (44.9) | 388 (48.7) | 504 (42.4) | 0.006 |
| Male | 1095 (55.1) | 409 (51.3) | 686 (57.6) |  |
| Race | | | | |
| White or Caucasian | 925 (46.6) | 302 (37.9) | 623 (52.4) | <0.001 |
| Black or African American | 708 (35.6) | 390 (48.9) | 318 (26.7) |  |
| Asian | 75 (3.8) | 18 (2.3) | 57 (4.8) |  |
| Other/Unknown | 279 (14.0) | 87 (10.9) | 192 (16.1) |  |
| Ethnicity | | | | |
| Hispanic/Latino | 315 (15.9) | 91 (11.4) | 224 (18.8) | <0.001 |
| Non-Hispanic/Latino | 1672 (84.1) | 706 (88.6) | 966 (81.2) |  |
| Severity of Illness Scores on Admission to the PICU | | | | |
| Phoenix Score | 0 [0,1] | 0 [0,1] | 0 [0,1] | 0.084 |
| pSOFA | 2 [1,4] | 2 [1,4] | 2 [1,4] | 0.002 |
| PELOD-2 | 3 [2,5] | 3 [2,5] | 3 [2,5] | 0.610 |
| PRISM III | 4 [1,6] | 4 [2,7] | 3 [0,6] | <0.001 |
| Length of Stay, days | | | | |
| Hospital | 2.4 [1.5,4.1] | 2.5 [1.4,4.4] | 2.3 [1.5,4.0] | 0.862 |
| PICU | 0.8 [0.6,0.9] | 0.8 [0.6,0.9] | 0.8 [0.7,0.9] | <0.001 |
| Outcomes | | | | |
| Sepsis | 216 (10.9) | 108 (13.6) | 108 (9.1) | 0.002 |
| Septic Shock | 119 (6.0) | 72 (9.0) | 47 (3.9) | <0.001 |
| In-hospital Mortality | 68 (3.4) | 42 (5.3) | 26 (2.2) | <0.001 |

Abbreviations: PELOD-2 - Pediatric Logistic Organ Dysfunction-2, PICU - Pediatric Intensive Care Unit, PRISM III - Pediatric Risk of Mortality III, pSOFA - Pediatric Sequential Organ Assessment.

*a* – P-values were computed using the Kruskal-Wallis test.
